# Assessment of Adequacy of Pain Management and Analgesic Use in Patients With Advanced Cancer Using the Brief Pain Inventory and Pain Management Index Calculation

**DOI:** 10.1200/JGO.2016.004663

**Published:** 2016-07-20

**Authors:** Harminder Singh, Raja Paramjeet Singh Banipal, Baltej Singh

**Affiliations:** All authors: Baba Farid University of Health Sciences, Faridkot, India.

## Abstract

**Purpose:**

The objective of this cross-sectional, noninterventional, 6-month observational study was to assess the adequacy of pain management in patients with cancer admitted to the Oncology Department of Guru Gobind Singh Medical College in Faridkot, India.

**Methods and Materials:**

A total of 348 patients with cancer were recruited for evaluation of the prevalence of inadequate cancer pain management using the Brief Pain Inventory Pain Management Index.

**Results:**

The current study included 127 males (36.5%) and 221 females (63.5%). The most prevalent cancer type was genitourinary; 268 patients (77%) had inadequately managed pain. A significant correlation was observed between poorly managed pain and age groups, analgesic used, and body mass index.

**Conclusion:**

Our observation of inadequate pain management among 77% of patients indicates that pain management was insufficient in three quarters of the patients in this study. Accumulating data regarding the inadequacy of cancer pain management is crucial to improve symptom management. Better management of pain not only alleviates pain symptoms but also increases the quality of life for patients with cancer.

## INTRODUCTION

Cancer prevalence in India is estimated to be approximately 2.0 to 2.5 million, with more than 0.7 to 0.8 million new cases identified every year.^1^ Currently, the state of Punjab is known as the cancer bowl of India, with a rising burden of cancer that leads to an additional problem of noncommunicable diseases.^2^

Cancer may manifest as a diverse range of signs and symptoms, such as unexplained weight loss, fever, fatigue, pain, and skin changes. However, pain is the most regular symptom of advanced cancer, increasing the emotional and physical challenges of patients.^3^ Conversely, appropriately managed pain or relatively controlled moderate or severe pain has been associated with amplified functionality and quality of life.^4^ Approximately 17% to 70% of patients with cancer experience pain at different stages of the disease; therefore, pain is an important health care problem for patients with cancer.^5^

The undertreatment of cancer pain is a well-known fact internationally, despite the existence of numerous guidelines for cancer pain management and wide-ranging consensus among health care professionals that 90% of patients with cancer can attain adequate pain relief with analgesics. Inadequate pain relief may depressingly impact a patient’s life. Health care providers, the patient’s family, and society play an important role in supporting patients who experience pain.^6-8^

The WHO developed guidelines for the treatment of cancer pain in 1986 (revised in 1996) that were aimed at decreasing the prevalence of inadequate analgesia.^9^ The guidelines contain suggestions about the type of analgesic that can be prescribed for pain that is normally mild, moderate, or severe. Mild pain should be managed with a nonsteroidal anti-inflammatory drug or acetaminophen. Weak opioids (eg, codeine) should be prescribed for moderate-level pain, and a strong opioid (eg, morphine, hydromorphone, oxycodone, fentanyl) should be prescribed for severe pain.^10^

Gauging the adequacy of pain management in cancer research is distinctly different from merely assessing pain intensity or pain relief because inadequacy is a predictor of functional impairment.^11,12^ Strictly following the WHO’s three-step analgesic treatment ladder is the best guideline in this regard.^9^ The situation is poor in developing countries such as India, where numerous challenges in optimum pain management have been described, such as a lack of reporting, poor communication, and misconceptions on the part of the patient and the health care staff regarding strong opioid use.^13^

Liberal use of opioids is always suggested for patients with advanced-stage cancer, but a conservative approach to opioid use may result in the undertreatment of cancer pain. With proper use of the WHO analgesic ladder, approximately 88% of patients reportedly obtain reasonable pain relief.^14^ The Pain Management Index (PMI) is a well-validated technique used to assess the adequacy of pain management on the basis of the WHO and Agency for Health Care Policy and Research guidelines; it is said to be a conservative measure and was developed by Cleeland et al^12,15^ for patients with cancer in 1994. To assess the adequacy of pain management in patients with cancer, we designed a study to estimate the prevalence of inadequately managed cancer pain by calculating PMI scores.

## METHODS AND MATERIALS

This was a cross-sectional, 6-month, noninterventional, prospective study conducted from January to July 2015 in the Oncology Department of Guru Gobind Singh Medical College, Faridkot, Punjab, India. A total of 348 patients with cancer participated in the study. All participants provided formal written consent, and ethical clearance was obtained from the institutional ethical review board. Patients were included in the study if they were diagnosed with cancer, were visiting the institution to receive chemotherapy, and agreed to participate. The exclusion criteria included a history of other chronic disease, such as diabetes or heart disease, and any known mental problem or being treated with psychotropic drugs.

Patients were interviewed, and sociodemographic variables, medical history, medication history, number of drugs prescribed (including analgesic medications), current diagnosis, and current medication information were collected on a case record sheet. Data on the PMI score, pain severity, pain interference with daily life, and adequacy of analgesic use were collected, along with the Brief Pain Inventory (BPI) score and visual analog scales to evaluate pain and its impact on daily function. The PMI score is a simple index that usually indicates how well the reported level of pain is managed by the analgesics prescribed. A pain score of 0 was defined as an absence of pain, 1 as mild pain, 2 as moderate pain, and 3 as severe pain. The BPI pain score categorization is 0 for an absence of pain, 1 to 4 for mild pain, 5 to 6 for moderate pain, and 7 to 10 for severe pain.^15,16^

A patient’s analgesic score on the PMI was calculated according to the type of analgesic prescribed by the physician. No prescribed analgesic was scored as 0, a nonopioid medication (ie, nonsteroidal anti-inflammatory drugs or acetaminophen) was scored as 1, a weak opioid (eg, codeine) was 2, and a strong opioid (eg, morphine, hydromorphone, oxycodone, fentanyl) as 3.^17^ The PMI calculation was then determined by subtracting the worst pain score from the analgesic score. Patients with negative PMI scores were classified as receiving inadequate analgesic treatment for their cancer pain.

Baseline distinctiveness (demographic, cancer-specific parameter) was summarized by descriptive statistics. Frequency, mean, percentages, and standard deviation were calculated wherever appropriate. Analyses to determine the association of patients’ adequately or inadequately managed pain with their age, sex, occupation, family history, duration, and cancer type were performed with appropriate statistical tests (analysis of variance and χ^2^ test). All *P* values ≤ .05 were considered significant.

## RESULTS

A total of 348 patients with cancer were included in this study; 127 (36.4%) were male and 221 (63.5%) were female. The mean age ± standard deviation was 52.12 ± 12.35 years (range, 28 to 85 years). The leading age group in the total study population was 40 to 50 years of age. The most prevalent cancer type was genitourinary, diagnosed in 100 patients (28.7%), which included cervical, prostate, ovarian, endometrial, and testicular cancer, followed by breast cancer in 80 patients (23%) and head and neck cancer in 71 patients (20.4%). The most common occupation was housewife (n = 218, 62.6%). A small number of patients (n = 16; 4.6%) presented with a positive family history of cancer (Table 1).

**Table 1:**
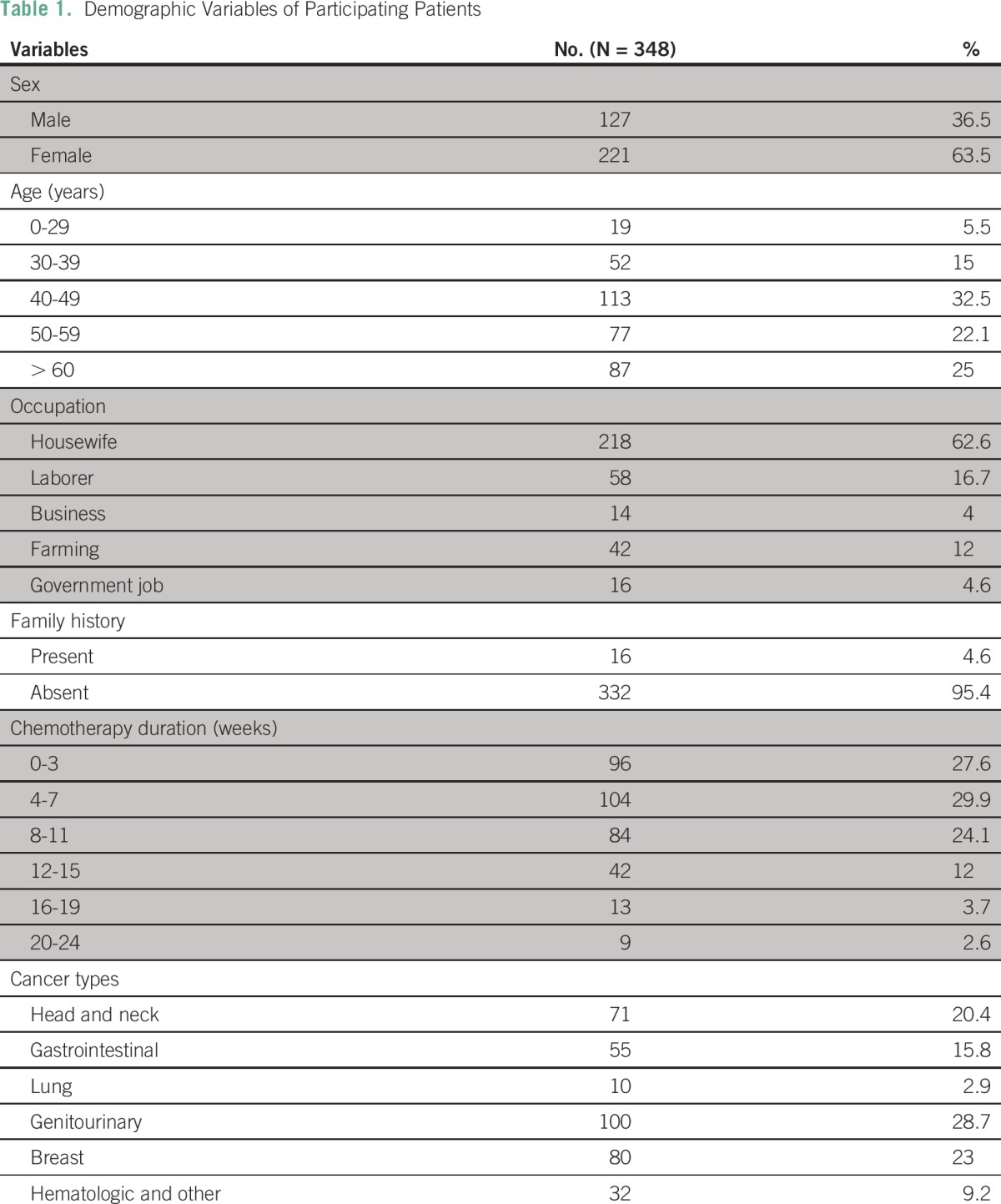
Demographic Variables of Participating Patients

Among the 348 patients, 268 (77%) had inadequate pain management/analgesia use, and 80 (23%) had better control of pain, as listed in Table 2. The association of adequate of pain management with the patient’s age, sex, occupation, family history, duration, cancer type, and body mass index (BMI) is listed in Table 2.

**Table 2:**
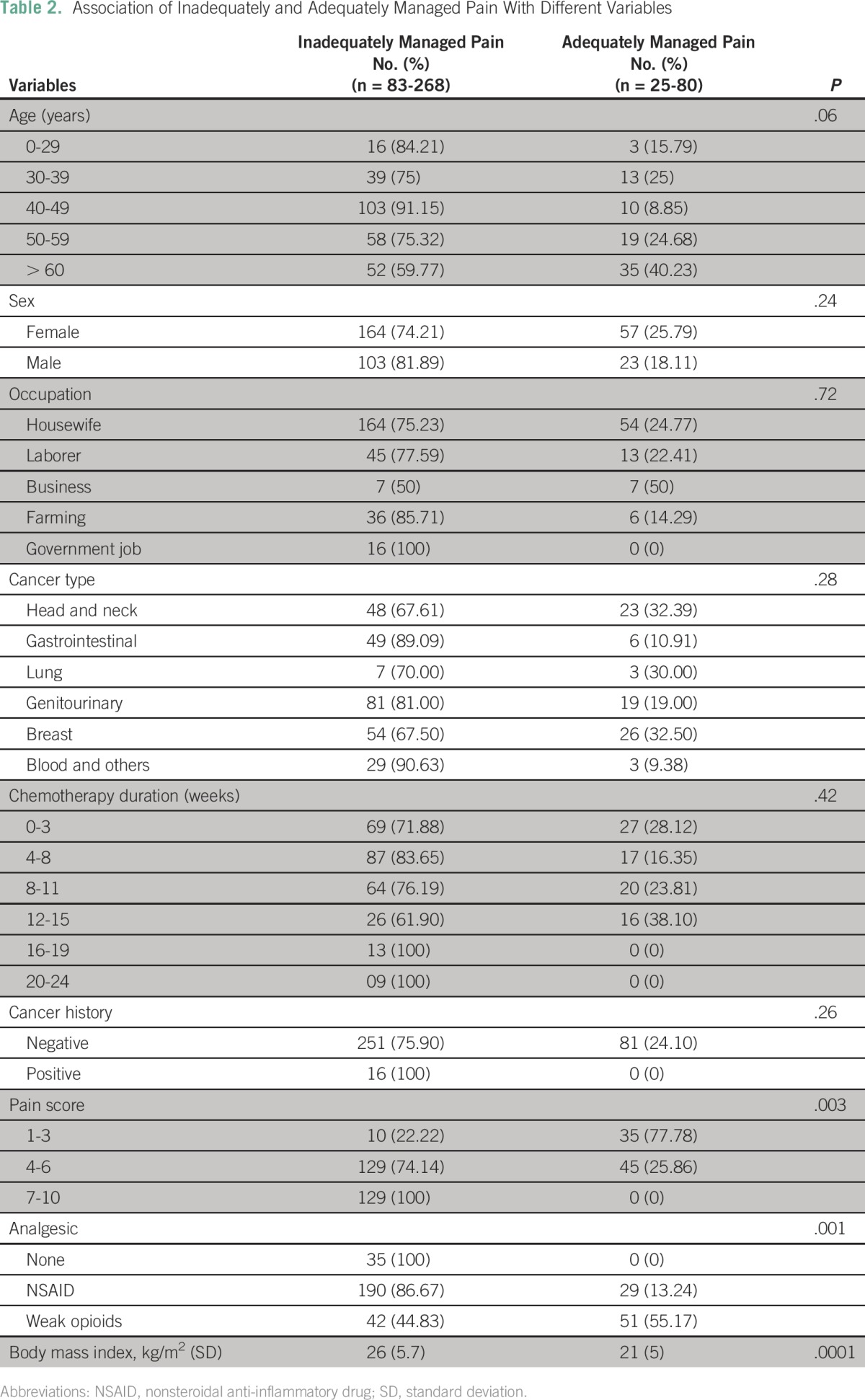
Association of Inadequately and Adequately Managed Pain With Different Variables

**Fig 1:**
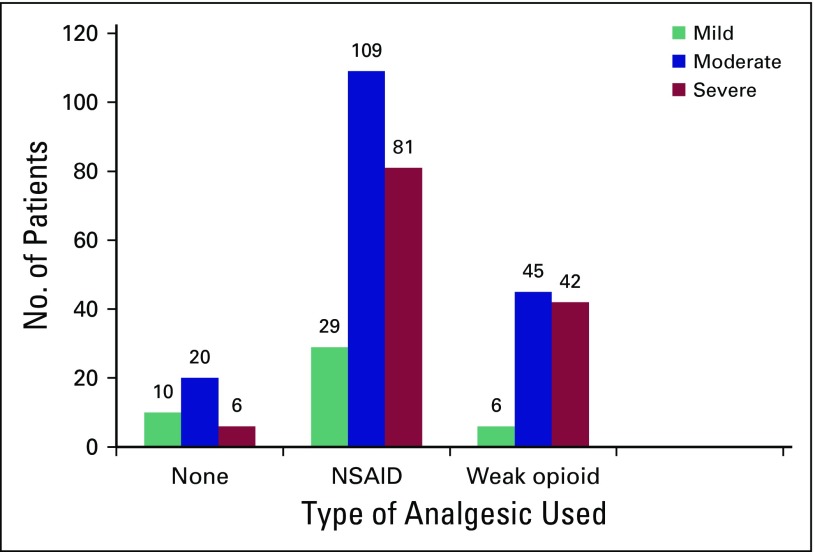
Analgesic use pattern in the 348 patients with cancer. NSAID, nonsteroidal anti-inflammatory drug.

The following groups had poor pain control: patients from 40 to 50 years of age (91.2%), males (81.9%) compared with females (74.21%), patients with genitourinary cancer (81%), patients taking nonsteroidal anti-inflammatory drugs (86.8%), and patients with higher BMI (obese), as listed in Table 2. No strong opioids were prescribed to any participants in the study (Table 2 and Fig 1).

BPI scores were used to assess patients’ painful experiences as a result of cancer (Table 3). According to the BPI severity score, 103 patients (29.6%) had mild pain, 226 (64.8%) had moderate pain, and 19 (5.6%) had severe pain. The BPI pain interference score showed that 277 patients (79.6%) experienced low pain interference in their day-to-day personal life and 71 (20.4%) experienced high pain interference. Figure 1 represents the analgesic use pattern in the 348 participants.

**Table 3:**
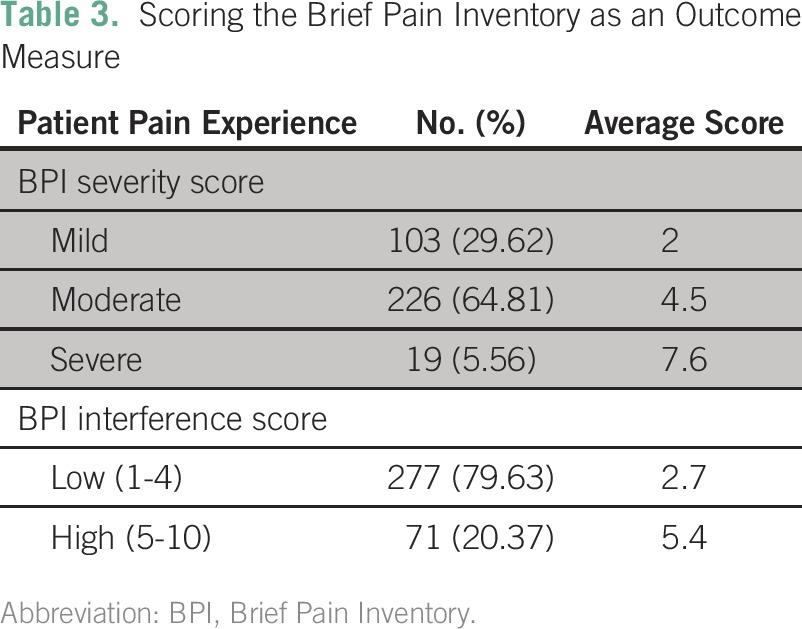
Scoring the Brief Pain Inventory as an Outcome Measure

## DISCUSSION

A high prevalence of inadequate pain management (77%) was found in our study population of 348 patients with cancer. The most important observation was that no strong opioids were prescribed to any of the patients in this study. In accordance with our experience, the majority of participants were concerned about their pain, regardless of the cause, stage of disease, treatment modality, or prognosis. For this reason, pain symptoms must be prevented, treated as a priority, and considered an independent part of cancer management.

This study examined the prevalence of inadequate analgesic treatment of patients with different types of cancer. Among the 348 patients in the study, approximately 77% were poorly managed or were undertreated for pain, which is a much higher proportion than that shown in the study by Kirou-Mauro et al,^18^ in which approximately 29% to 48% of patients were inadequately treated for their pain symptoms.

Few studies from developed countries have shown a lower number of undermedicated patients or patients whose pain was inadequately managed compared with the present study. A meta-analysis of 26 studies on undertreatment revealed that 43% of patients with cancer presented with a negative PMI.^15^ A US study by Cleeland et al^12^ found that 42% of patients were undermedicated. A European study showed that 57.5% of patients were undermedicated for cancer pain.^19^ Compared with developed countries, the prevalence of undermedication is higher in Asian countries. In China, a study by Wang et al^20^ showed that 67% of patients were undermedicated for cancer pain. A study in South Africa by Beck and Falkson^21^ reported that only 21% of patients with cancer had achieved 100% pain relief. A Canadian study by Vuong et al^22^ reported that 33.3% of patients reported inadequate pain management, and 106 of 354 patients reported severe pain despite taking strong opioids. In India, however, the proportion of inadequately managed pain was 79%, as reported by Saxena et al.^23^

The reason behind this disparity in cancer pain management between developed and underdeveloped countries is mainly that the affluent population of developed countries has relatively easy access to health care and required prescription drugs, and a culture exists in which taking pain medications is not perceived negatively.^24^ Furthermore, in many developing countries, morphine and other analgesics are not available or are not in regular supply, they might be expensive, or physicians are reluctant to prescribe them and patients are reluctant to use them because of known adverse effects and concern about addiction.

This study showed that female patients (25.8%) had better pain management than did male patients (18.1%). Simone et al^25^ found higher analgesic consumption rates among female participants. A study by Bernabei et al^26^ reported that the high level of well-managed pain in female patients with cancer may be attributed to their better knowledge about pain and pain management than men. In the current study, a higher BMI was related to poor pain control. A study by Stone et al^27^ showed a positive correlation between obesity and pain; explanations for this association include the effects of leptin and other hormones associated with excess fat and physiologic and psychologic factors associated with obesity, among others.

The Joint Commission on Accreditation of Healthcare Organizations^28^ in the United States developed a standard in 2001 that established new requirements for the assessment and management of cancer pain, including the patient’s right to appropriate assessment and management, follow-up, staff competency in pain management, policies supporting appropriate prescriptions, education of patients, and monitoring of appropriateness and effectiveness of pain management. All health care institutions providing cancer care must address this standard to provide better pain management.

In conclusion, the prevalence of inadequate pain management among 77% of the patients in our study was far too high and demonstrated that pain management was insufficient in more than three quarters of the patients. The undertreatment of pain-related cancer occurs worldwide and may fluctuate, depending on socioeconomic status, the patient’s culture, access to a doctor, the patient's inability to communicate the intensity of his or her pain, the reluctance of physicians to prescribe opioids, and patients’ reluctance to use opioids because of their known adverse events. Accumulating data regarding the inadequacy of cancer pain management is crucial to improve symptom management. We believe this study highlights the importance of cancer pain management and encourages providers to investigate the true status of cancer pain management. It also emphasizes the need for better education about pain and its control in the curricula of medical professionals. The systematic recording of pain intensity and follow-up of patients with cancer, regardless of the phase of the disease, should be routinely practiced in cancer wards. A better management approach will not only alleviate the pain symptoms but will also increase the patient’s quality of life.
